# Targeting AnxA1/Formyl Peptide Receptor 2 Pathway Affords Protection against Pathological Thrombo-Inflammation

**DOI:** 10.3390/cells9112473

**Published:** 2020-11-13

**Authors:** Shantel A. Vital, Elena Y. Senchenkova, Junaid Ansari, Felicity N. E. Gavins

**Affiliations:** 1 Department of Molecular and Cellular Physiology, Louisiana State University Health Sciences Center-Shreveport, Shreveport, LA 71130, USA; SVital@lsuhsc.edu (S.A.V.); ESench@lsuhsc.edu (E.Y.S.); junaid.em42@gmail.com (J.A.); 2 Department of Neurology, Louisiana State University Health Sciences Center-Shreveport, Shreveport, LA 71130, USA; 3 Department of Life Sciences, Centre for Inflammation Research and Translational Medicine (CIRTM), Brunel University London, Uxbridge, Middlesex UB8 3PH, UK

**Keywords:** thrombosis, inflammation, Annexin A1, formyl peptide receptors, sickle cell disease, sepsis

## Abstract

Stroke is a leading cause of death and disability globally and is associated with a number of co-morbidities including sepsis and sickle cell disease (SCD). Despite thrombo-inflammation underlying these co-morbidities, its pathogenesis remains complicated and drug discovery programs aimed at reducing and resolving the detrimental effects remain a major therapeutic challenge. The objective of this study was to assess whether the anti-inflammatory pro-resolving protein Annexin A1 (AnxA1) was able to reduce inflammation-induced thrombosis and suppress platelet activation and thrombus formation in the cerebral microvasculature. Using two distinct models of pathological thrombo-inflammation (lipopolysaccharide (LPS) and sickle transgenic mice (STM)), thrombosis was induced in the murine brain using photoactivation (light/dye) coupled with intravital microscopy. The heightened inflammation-induced microvascular thrombosis present in these two distinct thrombo-inflammatory models was inhibited significantly by the administration of AnxA1 mimetic peptide AnxA1_Ac2-26_ (an effect more pronounced in the SCD model vs. the endotoxin model) and mediated by the key resolution receptor, Fpr2/ALX. Furthermore, AnxA1_Ac2-26_ treatment was able to hamper platelet aggregation by reducing platelet stimulation and aggregation (by moderating α_IIb_β_3_ and P-selectin). These findings suggest that targeting the AnxA1/Fpr2/ALX pathway represents an attractive novel treatment strategy for resolving thrombo-inflammation, counteracting e.g., stroke in high-risk patient cohorts.

## 1. Introduction

Stroke is a leading cause of death and disability, with functional impairments producing significant losses in quality of life and accompanying financial burden [1,2,3]. Although the exact mechanisms responsible for post-ischaemic cerebral damage in stroke remain undefined, the intertwined processes of thrombosis and inflammation play crucial roles in the pathophysiology [4,5,6]. Unregulated thrombo-inflammation, which involves a complex relationship between inflammatory leukocytes (e.g., neutrophils), platelets, and the vascular endothelium, is also associated with a number of comorbidities such as sickle cell disease (“SCD” [7,8]) and infections (e.g., sepsis [9,10]), which predispose individuals to ischaemic stroke. In the case of SCD, ~11% of SCD patients have a stroke before the age of 20, increasing to 24% by the age of 45 [11]. Furthermore, stroke patients are not only more susceptible to bacterial infections, but infection itself is an independent risk factor for stroke and a major contributor to worse outcome post stroke, increasing recurrent stroke risk [4]. Therefore, reducing and resolving the impact and detrimental effects of microvascular thrombosis and inflammation associated with underlying co-morbidities, such as those already discussed, represent a major therapeutic challenge.

It is now widely accepted that endogenous pro-resolving mediators released during an inflammatory response play a critical role in effective recovery from inflammation and repair [12,13]. Resolution of inflammation is a tightly orchestrated process, involving specific endogenous mediators such as Annexin A1 (“AnxA1”) and its biologically active N-terminal domain, “Ac2-26” (Ac-AMVSEFLKQAWFIENEEQEYVQTVK); Lipoxins e.g., “Lipoxin A_4_” (5S,6R,15S-trihydroxy-7E,9E,11Z,13E-eicosatetraenoic acid) and aspirin-triggered lipoxin A_4_ (15(R)-epi-LXA_4_, “ATL”); resolvins (“Rv”) e.g., “RvD1” and “RvD2”; protectins e.g., protectin D1 (10R,17S-dihydroxy-4Z,7Z,11E,13E,15Z,19Z-docosahexaenoic acid); maresins e.g., maresin 1 (7R,14S-dihydroxy-4Z,8E,10E,12Z,16Z,19Z-docosahexaenoic, “MaR1”) [12,14,15,16]. These specific endogenous mediators moderate and resolve inflammation through protective pro-resolution pathways such as the formyl peptide receptor 2 (also termed “FPR2/ALX”, Fpr2, or ALX receptor i.e., the receptor for LXA_4_) pathway, which is a key resolution pathway [17,18,19,20,21,22].

The human FPR family consists of three specific receptors termed “FPR1”, FPR2/ALX, and “FPR3”, all of which are well conserved G-protein-coupled receptors expressed in a host of different cells and tissues, all having pluripotent and diverse roles in the initiation, propagation, and resolution of inflammation [17,19]. All three receptors are clustered together on chromosome 19q13.3 and share significant sequence homology, with FPR1 sharing ~69% and 56% sequence homology with FPR2/ALX and FPR3, respectively, and FPR2/ALX sharing 83% sequence homology with FPR3 [23].

AnxA1 is a 37 kDa protein that belongs to the annexin superfamily of Ca^2+^-dependent phospholipid binding proteins. It is an anti-inflammatory, pro-resolving protein that is up-regulated by glucocorticoids. Annexins share a common structure formed of a core region and a unique N-terminal domain, which acts as the fingerprint for each annexin of the superfamily. A number of investigations have focussed on the anti-inflammatory effects of AnxA1 and its mimetic peptides (including the blockade of leukocyte recruitment, inhibition of cytokine release, promotion of apoptosis, stimulation of phagocytosis, and decreasing vascular permeability) [17] in a variety of clinically related disease models [17,20,24,25,26,27,28], often via an engagement with FPR1 or FPR2/ALX (murine orthologues Fpr1 and Fpr2/ALX, respectively).

In the context of an ischaemic stroke setting, we have previously shown that AnxA1 (via Fpr2/ALX) is able to act both as a therapeutic and a prophylactic drug, reducing infarct volume and improving stroke outcome in a mouse model of acute experimental stroke [17,29] without increasing the risk of intracerebral haemorrhage [3,29,30]. Moreover, we demonstrated that this anti-inflammatory/pro-resolving compound was able to act as an anti-thrombotic agent suppressing thrombin-induced inside-out signalling events such as Akt activation, intracellular calcium release, and Ras-associated protein 1 (“Rap1”) [31], thereby exerting protection by altering platelet phenotypes from pro-pathogenic to regulatory [29]. These findings provided novel physio-pharmacological properties of AnxA1 as a powerful pro-resolving mediator of thrombo-inflammation and opened new avenues for an attractive therapeutic treatment strategy for patients with stroke. However, the anti-thrombotic effect of AnxA1 in patients with co-morbidities susceptible to stroke is less well characterized. Therefore, the objective of this current study was to use pharmacological and genetic approaches together with a photoactivation thrombosis (light/dye) model to test the hypothesis that targeting the AnxA1/FPR-pathway protects the cerebral microvascular system against inflammation-induced microvascular thrombosis in co-morbidities susceptible to stroke.

## 2. Materials and Methods

### 2.1. Animals

All studies were done blinded and performed on adult male mice weighing 25–30 g. C57BL/6 mice were purchased from Jackson Laboratory (Bar Harbor, ME, USA). The sickle cell transgenic mice (STM) (also termed β^s^ mice, heterozygous BERK, trait BERK, or sickle cell trait model) were homozygous for knockout of murine α-globin and heterozygous for knockout of murine β-globin and had one copy of the linked transgenes for human α- and β^s^-globins [32]. STM used in this study were a gift from Professor Robert Hebbel (University of Minnesota). STM were bred onsite and showed no obvious phenotype and were fertile. Mice were maintained on a 12 h (h) light–dark cycle, during which room temperature was maintained at 21–23 °C. Mice had access to a standard chow pellet diet and tap water *ad libitum*. All animal experiments were approved by the Louisiana State University Health Sciences Center—Shreveport (LSUHSC-S) Institutional Animal Care and Use Committee (IACUC), were in accordance with the guidelines of the American Physiological Society and complied with ARRIVE (Animal Research: Reporting In Vivo Experiments) guidelines.

### 2.2. Endotoxin (LPS) Administration

Mice were injected intraperitoneally (i.p.) with “LPS” (Escherichia coli serotype 0111:B4, purified initially by phenol extraction and purified further by ion exchange chromatography. L3024. Sigma-Aldrich, St Louis, MO, USA), two hours before the experiment at a dose of 0.4 mg/kg in 100 μL vehicle sterile saline [33].

### 2.3. Photoactivation Thrombosis Model. (Light/Dye Method)

*Intravital microscopy (“IVM”)* was performed as previously described [29]. Briefly, mice were anaesthetized via i.p. injection of ketamine (150 mg/kg) and xylazine (7.5 mg/kg), and the femoral vein was cannulated for dye (fluorescein isothiocyanate (FITC)-dextran) administration. The head of each mouse was fixed in a frame (sphinx position) and the parietal bone was exposed by a midline skin incision, followed by a craniectomy [29]. A total of 10 mg/kg of 5% FITC dextran (150,000 mol wt, Sigma-Aldrich) was injected i.v. and allowed to circulate for ten minutes (mins) prior to photoactivation. Photoactivation was initiated (excitation, 495 nm; emission, 519 nm) by exposing 100 μm of vessel length to epi-illumination with a 175-W xenon lamp (Lambda LS, Sutter, Novato, CA, USA) and a fluorescein filter cube (HQ-FITC, Chroma, San Francisco, CA, USA) [29]. The excitation power density was measured daily (ILT 1700 Radiometer, SED033 detector; International Light, Peabody, MA, USA) and maintained within 1% of 0.77 W/cm^2^. Epi-illumination was applied continuously, and onset and blood flow cessation times were quantified in cerebral vessels (diameter range: 30–70 μm) until blood flow had ceased in the vessel under study [29]. In some cases, 20 min prior to onset of thrombosis, mice were treated with vehicle (saline), AnxA1_Ac2-26_ (Ac-AMVSEFLKQAWFIENEEQEYVQTVK, 4 mg/kg. Cambridge Research Biochemicals, Cambridge, UK), pan FPR antagonist Boc2 (N-tert-butoxycarbonyl-L-Phe-D-Leu-L-Phe-D-Leu-L-Phe, 0.4 mg/kg; MP Biomedicals, Cambridge, UK) or FPR2/ALX antagonist WRW4 (2.2 mg/kg. Tocris, Bristol, UK) administered (100 µL) i.v. [20,29]. Doses/concentrations used in the study are congruent with binding affinities for the Fprs and were chosen based on our previous findings [17,20,29]. All compounds were made in vehicle sterile saline.

### 2.4. Bleeding Time

Bleeding times were quantified in mice treated with vehicle saline, LPS, or AnxA1_Ac2-26_, as previously described. Briefly, mice were anaesthetized with i.p. injection of ketamine (150 mg/kg) and xylazine (7.5 mg/kg). A small tail segment (3 mm) was cut cleanly with a scalpel blade, and bleeding was monitored at 15-s intervals by absorbing the bead of blood with filter paper without contacting the wound site [29]. When no blood was observed on the paper, bleeding was determined to have ceased [34].

### 2.5. Platelet Cell Counts

Platelets from peripheral blood were stained with 1% buffered ammonium oxalate and counted using a haemocytometer.

### 2.6. Platelet Flow Cytometry

Whole blood was collected (0.9 mL) via carotid artery into a syringe containing 0.1 mL anticoagulant citrate dextrose (“ACD”) buffer, transferred into an Eppendorf tube, and centrifuged at 1200 rpm for 8 min. The platelet-rich plasma (“PRP”) layer was transferred to a new Eppendorf tube and centrifuged at 3000 rpm for 10 min. The supernatant was removed and the pellet was resuspended in Tyrodes buffer (containing 1 mM Ca^2+^) along with the appropriate antibody and allowed to incubate for 15 min at 37 °C. The reaction was stopped by the addition of 450 μL of 1% paraformaldehyde. P-selectin and JON/A-PE FITC antibodies (Emfret Analytics, Eibelstadt, Germany) were used to measure platelet activation and surface P-selectin exposure in murine platelets using flow cytometry, as previously described [35,36]. IgG isotype antibodies were used as controls. Briefly, the platelets were treated with Fc block (15 min, room temperature) to inhibit non-specific binding according to the manufacturer’s instructions (eBioscience, San Diego, CA, USA). This was followed by treatment with AnxA1_Ac2-26_ (30 μM) or vehicle for 30 min and then, the addition of antibodies (1:8 dilution). Next, the glycoprotein VI (GPVI) collagen receptor agonist convulxin (CVX) (1.7 ng/mL. Cayman Chemical Company, MI, USA) was used to stimulate the platelets for 15 min. In some cases, prior to CVX, platelets were treated with LPS (7.5 μg/mL). The activation was stopped by the addition of 450 μL of 1% paraformaldehyde. Platelets were identified by their light scattering using an LSRII flow cytometer (Becton Dickinson, Franklin Lakes, NJ, USA) and Diva8 software by assessing at least 10,000 events per sample, as previously described [34].

### 2.7. Platelet Aggregation Assay

Arterial blood was freshly collected from the carotid artery of mice, as described above. Platelets 8–10* × *10^6^/mL were used to monitor platelet aggregation velocity after agonist exposure in a cuvette loaded with 6 mL of platelet media (140 mM NaCl, 10 mM HEPES, 10 mM NaHCO_3_, 2 mM KCl, 1 mM MgCl_2_, 2 mM CaCl_2_, 5.5 mM D-glucose, pH 7.4) using a laser particle analyser (LasCa-1C, Lumex Ltd., St. Petersburg, Russia) by a low angle light scattering method, as previously described [29]. Platelets were administered either vehicle (saline) or LPS (7.5 μg/mL) and the normalized velocity of aggregation was calculated using original software LasCa_32, as previously described [29].

### 2.8. Statistical Analysis

All data were analysed using GraphPad Prism8 software. Data are shown as mean values ± standard error of the mean (SEM), with *n* values given in the respective figures. Results from thrombosis experiments were confirmed to follow a normal distribution using a Kolmogorov–Smirnov test of normality with Dallal–Wilkinson–Lillie for the corrected *p* value. Data that passed the normality assumption were analysed using Student’s *t*-test (two groups) or ANOVA with Bonferroni post hoc tests (more than two groups). Data that failed the normality assumption were analysed using the non-parametric Mann–Whitney U test (two groups) or Kruskal–Wallis with Dunn’s test (more than two groups). Differences were considered statistically significant at a value of *p* < 0.05.

## 3. Results

### 3.1. AnxA1_Ac2-26_ Inhibits LPS-Induced Thrombus Formation in Cerebral Microcirculation of C57/BL6 Mice

**Figure 1:**
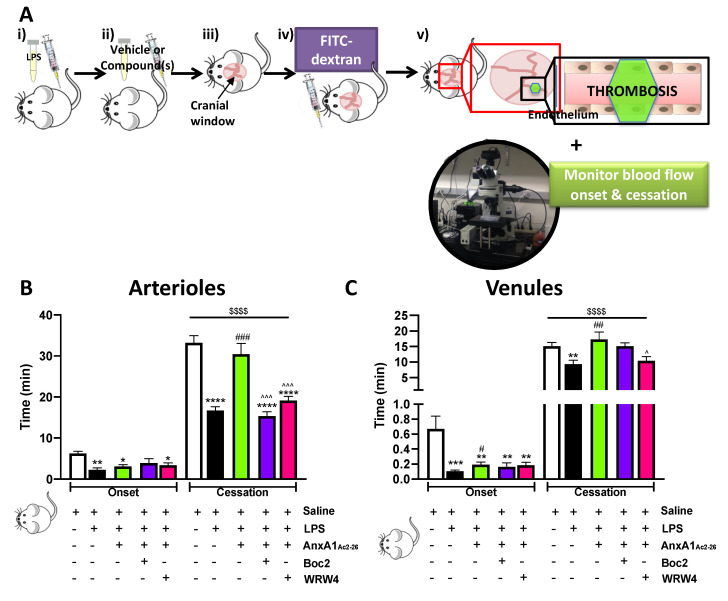
AnxA1_Ac2-26_ protects against the effects of endotoxin (LPS) in light/dye-induced thrombosis responses in the cerebral microcirculation. (**A**) Schematic showing the experimental protocol: (i) Mice (C57BL/6) were subjected to vehicle (saline) or LPS (0.4 mg/kg) for 2 h. (ii) Mice were treated with vehicle (saline) or compound(s): AnxA1_Ac2-26_ (4 mg/kg) with/without pan FPR antagonist Boc2 (0.4 mg/kg) or FPR2/ALX antagonist WRW4 (2.2 mg/kg) 20 min prior to thrombosis. (iii) A cranial window was performed and (iv) fluorescein isothiocyanate (FITC)-dextran was injected (10 mg/kg of 5%). (v) Mice were subjected to intravital microscopy and light/dye-induced thrombus formation, with onset and blood flow cessation times recorded for cerebral (**B**) arterioles and (**C**) venules. Data are means ± SEM of 5–6 mice/group. * *p* < 0.05, ** *p* < 0.01, *** *p* < 0.001, **** *p* < 0.0001 vs. corresponding vehicle (saline). ^#^ *p* < 0.05, ^##^ *p* < 0.01, ^###^ *p* < 0.001 vs. corresponding LPS group. ^$$$$^ *p* < 0.0001 vs. same group for onset time. ^ *p* < 0.05, ^^^ *p* < 0.001 vs. corresponding LPS+AnxA1_Ac2-26_ group.

The light/dye model coupled with intravital microscopy was used to quantify thrombus formation in cerebral arterioles and venules, as determined by onset time (i.e., the time to onset of visible aggregates) and blood flow cessation time (Figure 1A). Figure 1B,C shows that onset time was significantly faster in both arterioles and venules in LPS-treated mice vs. saline (vehicle)-treated mice (with the exception of LPS+AnxA1_Ac2-26_+Boc2). We found that administration of AnxA1_Ac2-26_ delayed the time of onset in the venules of mice treated with LPS vs. LPS alone (*p* < 0.05), suggesting a propensity for the AnxA1 peptide to initiate an early anti-thrombo-inflammatory response against inflammation-induced thrombosis.

As previously reported in C57BL/6 mice [34], LPS treatment augmented thrombus formation in the cerebral microcirculation (as noted by the decreased blood flow cessation times in LPS-treated mice vs. their saline-treated counterparts). This pro-thrombotic environment was mitigated by the treatment of AnxA1_Ac2-26_, which was effective in both vessel types analysed (arterioles: 16.7 ± 1.0 min vs. 30.5 ± 2.7 min; venules: 9.3 ± 1.3 min vs. 17.3 ± 2.4 min; LPS vs. LPS+ AnxA1_Ac2-26_, respectively).

Having established the anti-thrombotic effect of AnxA1_Ac2-26_ against inflammation-induced thrombosis, we next ascertained whether these effects were mediated by the classic AnxA1/Fpr pathway. Figure 1B,C shows the FPR pan-antagonist Boc2 inhibited the actions of the AnxA1 mimetic peptide (*p* < 0.0001) in cerebral arterioles, but not in venules, suggesting a vessel-specific mechanism of action via the FPR family. (No effect was observed in either onset or blood flow cessation times for Boc2 administration alone vs. LPS; see Figure S1).

To further expand on these findings and tease out through which member(s) of the FPR family AnxA1_Ac2-26_ was exerting its protective effects, mice were co-administered AnxA1_Ac2-26_ with WRW4 (specific FPR2/ALX antagonist). We found that WRW4 blocked AnxA1_Ac2-26_ afforded protection in both cerebral arterioles (*p* < 0.001) and venules (*p* < 0.05), thereby confirming an FPR2/ALX mechanism (Figure 1B,C). (Figure S1 shows that no effect was noted in either onset or blood flow cessation times for administration of WRW4 alone vs. LPS). These data further support the growing body of evidence that the FPR2/ALX pathway plays a crucial role in both thrombotic and inflammatory pathways in the brain microvasculature.

### 3.2. AnxA1_Ac2-26_ Reduces the Effect of Endotoxin-Induced Platelet Activation

**Figure 2:**
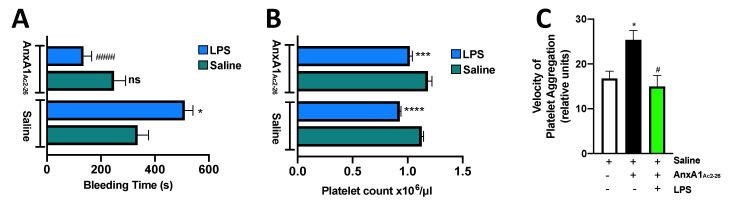
AnxA1_Ac2-26_ treatment inhibits endotoxin-induced activation of the platelet activation. (**A**) tail bleeding times and (**B**) peripheral blood platelet counts were assessed following saline (vehicle) or AnxA1_Ac2-26_ (4 mg/kg) administration for 20 min following 2 h saline (vehicle) or LPS (0.4 mg/kg) administration. (**C**) Isolated platelets were treated with saline or LPS (7.5 μg/mL) with or without AnxA1_Ac2-26_ (1 μg/mL) and the velocity of aggregate formation was measured using a low-angle light-scattering technique. Data are means ± SEM of 5–6 mice/group. * p < 0.05, *** p < 0.001, **** p < 0.0001 vs. corresponding vehicle (saline). ^#^ *p* < 0.05, ^####^ *p* < 0.0001 vs. corresponding LPS group. ns—non-significant vs. corresponding saline group.

Since AnxA1_Ac2-26_ was able to reduce thrombosis, we next sought to determine whether exogenous administration of AnxA1_Ac2-26_ had any effect on the haemostatic activity of platelets. Figure 2A shows that AnxA1_Ac2-26_-treated LPS mice exhibited decreased bleeding times (*p* < 0.001), and these observations were not related to changes in circulating platelet cell counts as AnxA1_Ac2-26_ administration had no effect on the LPS-heightened platelet cell counts (Figure 2B). The interaction of platelets with neutrophils is involved in many thrombo-inflammatory responses. Furthermore, LPS is a potential mediator of neutrophil extracellular traps (NETosis) and induces platelet–neutrophil aggregate formation, and neutrophils have been shown to be essential for platelet recruitment in endotoxaemic models [37,38]. Here, we found that circulating neutrophil counts were heightened in those mice challenged with LPS, but these levels were not modified by AnxA1_Ac2-26_ (Figure S3). We also found that the peptide was able to act as an anti-aggregant in its ability to moderate platelet aggregation induced by LPS (by reducing the velocity of platelet–platelet aggregate formation; see Figure 2C). These data provide further evidence that not only is the AnxA1 mimetic peptide a known anti-inflammatory drug, but it also has potential as an effective anti-platelet drug.

### 3.3. AnxA1_Ac2-26_ Affords Protection against Cerebral Thrombo-Inflammation

**Figure 3:**
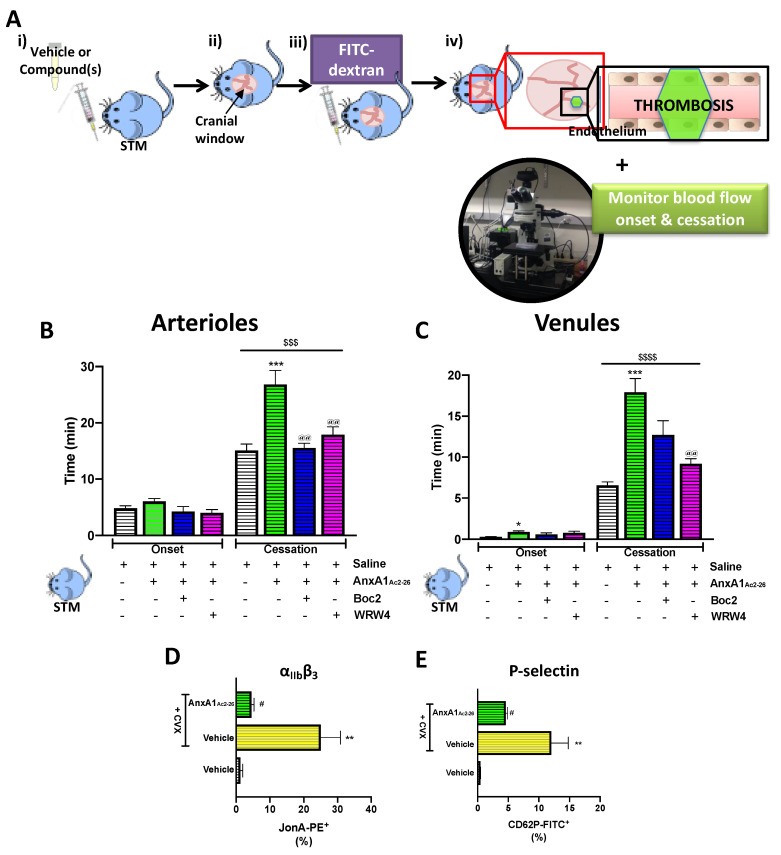
AnxA1_Ac2-26_ affords protection against cerebral thrombo-inflammation in STM and modifies cell surface expression of platelet receptors. (**A**) Schematic showing the experimental protocol: (i) sickle cell transgenic mice (STM) were treated with vehicle (saline) or compound(s): AnxA1_Ac2-26_ (4 mg/kg) with/without pan FPR antagonist Boc2 (0.4 mg/kg) or FPR2/ALX antagonist WRW4 (2.2 mg/kg) 20 min (mins) prior to thrombosis. (ii) A cranial window was performed and (iii) FITC-dextran was injected (10 mg/kg of 5%) and allowed to circulate for 10 min before STMs were subjected to (iv) intravital microscopy and light/dye-induced thrombus formation. Onset time and blood flow cessation times were recorded for cerebral. (**B**) arterioles and (**C**) venules. (**D+E**) Activated integrin α_IIb_β_3_ and P-selectin were quantified using flow cytometry in platelets isolated from STM following 15 min stimulation with GPVI collagen receptor agonist, convulxin (CVX, 1.7 ng/mL). Vehicle (saline) or AnxA1_Ac2-26_ (30 μM) was given 30 min prior to activation, followed by vehicle (saline) or LPS (7.5 μg/mL for 5 min). (**D**) % of activated integrin α_IIb_β_3_ (JON-A) and (**E**) % of activated P-selectin expression (CD62P). Data are means ± SEM of 5–6 mice/group. * *p* < 0.05, ** *p* < 0.01, *** *p* < 0.001 vs. corresponding vehicle (saline). ^@@^ *p* < 0.01 vs. corresponding Ac2-26 group. ^$$$^ *p* < 0.001, ^$$$$^ *p* < 0.0001 vs. same group for onset time. ^#^ *p* < 0.05 vs. CVX+vehicle group.

Having assessed the anti-thrombotic effect of AnxA1_Ac2-26_ on endotoxin (LPS)-enhanced thrombosis, a model which is known to favour venular thrombosis [39], we next wanted to determine whether the protective effect of the peptide was model-specific. SCD assumes a pro-inflammatory and pro-thrombotic phenotype throughout the microvasculature. As such, we performed the light/dye thrombosis model in STM, which are known to share these clinical features [32,40,41]. Figure 3 shows that without stimulation, STM assume a pro-thrombotic phenotype, as evidenced by much quicker (~50% quicker) blood flow cessation times than non-STM (Figure S2) in both sides of the vascular tree (arterioles: 33.2 ± 1.8 min vs. 15.1 ± 1.2 min (*p* < 0.05); venules: 15.1 ± 1.2 vs. 6.5 ± 0.4 (*p* < 0.05); non-STM vs. STM). Moreover, in comparison to saline-treated STM, administration of AnxA1_Ac2-26_ significantly protected (*p* < 0.01) against thrombotic events by causing a significant increase in blood flow in arterioles by 47.8% and in venules by 63.5%, respectively, an effect that was again mitigated by the co-administration of either Boc2 or WRW4 (Figure 3B,C). (No differences were observed in blood flow onset times when either of the two antagonists were administered alone; see Figure S4). Interestingly, AnxA1_Ac2-26_ also delayed the onset time in venules (Figure 3B), an effect that concurred with the effect on cerebral venules following LPS-induced thrombosis (Figure S2).

### 3.4. AnxA1_Ac2-26_ Primes Platelet Activation via GPVI Pathway Regulation in SCD-Associated Thrombo-Inflammation

Having found that AnxA1_Ac2-26_ was able to modify the thrombo-inflammatory environment by prolonging blood flow cessation in the cerebral microcirculation in both arterioles and venules, we next sought to investigate the influence of this AnxA1 mimetic peptide on a key platelet receptor which is known to play a central role in thrombosis, i.e., GPVI. This receptor is able to stimulate platelet adhesion by its ability to enhance the affinity of other integrins such as α_IIb_β_3_ via inside-out signalling mechanisms [42]. Figure 3D,E show that stimulation of platelets with the GPVI collagen receptor agonist CVX potentiated both α_IIb_β_3_ (Figure 3C) and P-selectin in STM (Figure 3D). Direct treatment of platelets with AnxA1_Ac2-26_ (30 μM) was found to suppress these levels (*p* < 0.05); although the effects on P-selectin expression were significant, they were less dramatic as those observed with α_IIb_β_3_. Taken together, these findings suggest that AnxA1_Ac2-26_ is able to reduce the susceptibility of platelets to interact with collagen receptors, thereby reducing the propensity for platelets to aggregate and cause thrombosis in a thrombo-inflammatory environment.

### 3.5. Exploiting the AnxA1/FPR2/ALX Pathway as a Therapeutic Strategy to Alleviate Thrombo-Inflammation

**Figure 4:**
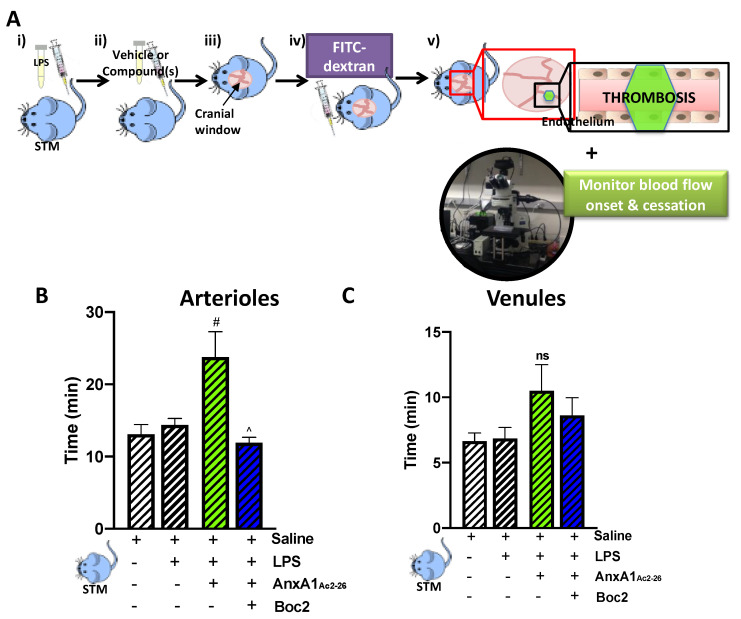
AnxA1_Ac2-26_ affords arteriolar protection against cerebral inflammation-induced thrombosis in sickle cell transgenic mice (STM). (**A**) schematic showing the experimental protocol: (i) STM were subjected to vehicle (saline) or LPS (0.4 mg/kg) for 2 h and (ii) treated with vehicle (saline) or compound(s): AnxA1_Ac2-26_ (4 mg/kg) with/without pan FPR antagonist Boc2 (0.4 mg/kg) 20 min prior to thrombosis. (iii) A cranial window was performed and (iv) FITC-dextran was injected (10 mg/kg of 5%). (v) Mice were subjected to intravital microscopy and light/dye-induced thrombus formation, with onset and blood flow cessation times recorded for cerebral (**B**) arterioles and (**C**) venules. Data are means ± SEM of 5–6 mice/group. ^#^ *p* < 0.05 vs. LPS group. ^ *p* < 0.05, vs. LPS+AnxA1_Ac2-26_ group. ns—non-significant vs. corresponding LPS group.

A pro-inflammatory disease state exists in SCD, which predisposes patients to vaso-occlusion (VOC) in response to triggering factors such as infection [43], thus heightening the risk for stroke [11], acute chest syndrome [44], and early death [45]. As such, in the final part of the study, we validated the therapeutic potential of AnxA1_Ac2-26_ as a treatment therapy against thrombo-inflammation in STM following LPS injection and photoactivation to induce a VOC (Figure 4A). Markedly, STM mice treated with LPS did not display an additive thrombo-inflammatory response (Figure 4B,C) above that observed in the absence of LPS (Figure 3B,C), suggesting an already exhaustive SCD phenotype. Nonetheless, Figure 4B shows that STM mice treated with AnxA1_Ac2-26_ caused a prolongation of blood flow cessation responses in arterioles, which was abrogated in the presence of Boc2. This protective effect afforded by the peptide was not mirrored in cerebral venules, despite there being a trend towards an increase (Figure 4C).

## 4. Discussion

**Figure 5:**
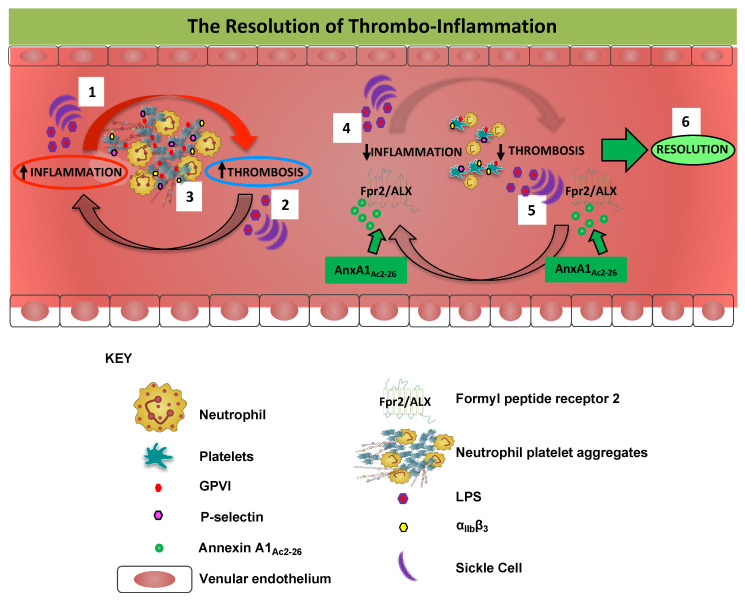
Anti-inflammatory and pro-resolving effects of targeting the AnxA1/Fpr2/ALX pathway against thrombo-inflammation. Thrombo-inflammatory conditions such as sepsis [35] and sickle cell disease (SCD) [71] induce inflammation (Box 1) as well as thrombus formation (Box 2) in the cerebral microcirculation enabling thrombo-inflammation (as indicated by e.g., heightened platelet GPVI, α_IIb_β_3_ and P-selectin expression, and increased platelet–platelet (homotypic) aggregates and platelet-neutrophil (heterotypic) aggregates). Neutrophils also produce neutrophil extracellular traps (NETs) which can exacerbate thrombo-inflammation (Box 3). AnxA1_Ac2-26_ inhibits lipopolysaccharide (LPS) and SCD-induced cerebral inflammation (Box 4) and cerebral thrombosis (Box 5) via Fpr2/ALX. The AnxA1 peptide affords protection by altering the haemostatic action of platelets, modulating platelet cell surface molecules elicited by GPVI (e.g., α_IIb_β_3_ and P-selectin), and reducing platelet (homotypic and heterotypic) aggregation. Thus, AnxA1_Ac2-26_ promotes the resolution of thrombo-inflammation by reducing platelet activation, adhesion, and aggregation which cause and promote thrombosis (Box 6).

The interplay between thrombosis and inflammation (thrombo-inflammation) occurs in a broad range of human disorders (including stroke, sepsis, and SCD), with ensuing complications transpiring to be more hazardous in the microvasculature of injured tissues and organs [46]. Given the devastating impact of pathological thrombo-inflammation, there is an unmet clinical need to understand the complex pathophysiology for therapeutic development of drugs that are more efficacious, have fewer side-effects, and are devoid of bleeding complications that would ultimately undermine the clinical benefit. Here, using pharmacological and genetic approaches together with a photoactivation thrombosis (light/dye) model coupled with intravital microscopy, we provide further evidence of the multifaceted role of AnxA1 N-terminal mimetic peptide AnxA1_Ac2-26_ as an anti-thrombotic and anti-coagulant agent. Our data demonstrate that AnxA1_Ac2-26_ affected not only the haemostatic action of platelets (e.g., reduced bleeding times), but moderated platelet aggregation (e.g., diminished the propensity for platelets to form platelet–platelet aggregates) and lowered thrombogenesis (i.e., reduced the time of onset of platelet deposition/aggregation within cerebral vessels), thereby reducing LPS-induced thrombosis. Furthermore, the AnxA1 peptide was able to decrease α_IIb_β_3_ activation and reduce P-selectin expression elicited by GPVI, inhibiting platelet activation and thrombosis in STM. Taken together, our data reveal that AnxA1_Ac2-26_ affords protection by altering the haemostatic action of platelets, modulating platelet cell surface molecules (e.g., α_IIb_β_3_ and P-selectin), and reducing platelet heterotypic aggregation. In so doing, AnxA1_Ac2-26_ reduces platelet activation, adhesion, and aggregation and tempers thrombosis. Collectively, these results highlight the potential for AnxA1_Ac2-26_ as a viable therapy for the management of thrombo-inflammatory disorders (as shown in Figure 5).

The resolution of inflammation is a tightly orchestrated process that is controlled by endogenous biosynthetic mediators such as AnxA1 and its mimetic peptide (AnxA1_Ac2-26_). Both these compounds have been shown to act at various points in the inflammation-resolution pathway [47]. The known anti-inflammatory actions of AnxA1_Ac2-26_ (e.g., inhibiting neutrophil recruitment, decreasing neutrophil endothelium interactions, and suppressing inflammatory cytokine production [47]) have been widely studied and these actions eventually contribute to inflammation resolution by enabling apoptosis [14] and phagocytosis [29]. Previously, it was thought that inflammation and thrombosis were two independent processes. However, the innate and coagulation systems are so intertwined that these two processes are now considered to be in part the same, in that microvascular thrombosis is accompanied by inflammation and vice versa, an association referred to as thrombo-inflammation [17]. However, a greater understanding of the links between inflammation and thrombosis is needed in order to develop new therapeutic opportunities [29]. Our laboratory is actively pursuing therapeutic drug discovery programs focused on the concept of thrombo-inflammation resolution, with the AnxA1/FPR2/ALX pathway being of significance.

It is estimated that >80% of sepsis patients have either clinical or subclinical hypercoagulopathy, increasing the risks for both thrombosis and haemorrhage [48,49]. These increased thrombotic risks can function not only as acute triggers for stroke but are associated with poor long-term prognosis after stroke [48]. Endotoxin (LPS) is a component of Gram-negative bacteria cell walls, producing many clinical manifestations of Gram-negative sepsis [50]. Using an animal model of endotoxaemia, we found AnxA1_Ac2-26_ treatment counteracted the enhanced microvascular thrombus formation, an effect which was found to be mediated through Fpr2/ALX. These protective effects are in line with the defensive actions of AnxA1_Ac2-26_ on the inflammatory cascade that have been observed previously in the microcirculation post-LPS challenge. In these studies, AnxA1_Ac2-26_ was able to abrogate leukocyte adhesion and plasma protein extravasation in the brain [33] and the mesentery. Furthermore, AnxA1 and AnxA1_Ac2-26_ both cause leukocyte detachment, reducing the inflammatory environment and driving resolution [51]. Within this study, we have found that although AnxA1_Ac2-26_ treatment prolonged blood flow cessation time within a thrombotic LPS environment, it also lowered thrombogenesis (i.e., reduced the time of onset of platelet deposition/aggregation within cerebral vessels). Previously, we have shown that whole protein AnxA1 treatment reduces platelet adhesion to the cerebral endothelium following cerebral I/RI [17]. However, although thrombosis and inflammation are interlinked processes, the lack of effect on platelet adhesion observed here may relate to the type of model (thrombotic vs. inflammatory) and/or the possibility that, as with the parent compound in cerebral I/RI, AnxA1_Ac2-26_ is orchestrating a complex change in the platelet phenotype from pro-pathogenic to regulatory (which concurs in cerebral I/RI [29]) and in doing so, enhances blood flow cessation times.

In the clinic, the pro-thrombogenic phenotype observed in sepsis patients is often accompanied by thrombocytopenia (with prolonged time span of thrombocytopenia being correlated with increased mortality in intensive care patients [52]), reactivity of platelets and by an imbalance between procoagulant and anticoagulant mechanisms [49]. Thus, platelet count is a useful marker of adverse disease activity, although it remains unclear as to whether the reduction in the number of platelets is mechanistically linked to heightened platelet activity and increased thrombosis. Here, LPS treatment caused thrombocytopenia which concurred with previous findings, not only by our group [53], but also by others e.g., George et al. showed LPS to induce severe thrombocytopenia and inflammation resulting in spontaneous intra-alveolar haemorrhage [54] and Hillgruber et al. concluded that thrombocytopenia in the skin and lungs can be limited by targeting neutrophil diapedesis through the endothelial barrier [55], demonstrating the importance of the cross-talk between neutrophils and platelets. In our study, although AnxA1_Ac2-26_ did not affect thrombocytopenia induced by LPS (which may relate to the mechanisms that regulate thrombosis and haemostasis), it did reduce bleeding time in an in vivo assay that is widely used to assess the haemostatic action of platelets [56]. These findings are of clinical significance because the efficacy of anti-platelet agents is often limited by bleeding complications. Genetic *AnxA1* deletion is not associated with spontaneous thrombosis or bleeding but leads to exacerbated cerebral inflammatory responses following experimental ischaemic stroke [29]. Furthermore, decreased circulating levels of AnxA1 are present in thrombo-inflammatory conditions including ischaemic stroke, SCD, sepsis, Crohn’s disease, and obesity [29]. Taken together, these results demonstrate AnxA1_Ac2-26_ attenuates LPS-induced peripheral bleeding and intravascular thrombosis, affording a therapeutic strategy for thrombotic complications.

Defining molecular mechanisms regulating thrombo-inflammation in specific disease states is of major clinical importance. Having characterised the effects of AnxA1_Ac2-26_ on inflammation-induced thrombosis instigated by LPS, we next wanted to ascertain whether the protective effects elicited by the AnxA1 peptide were specific to the LPS experimental model of thrombo-inflammation. As such, we focused on SCD, an inherited autosomal recessive disorder (resulting from a single amino acid substitution in the haemoglobin β chain) whose pathophysiology is characterized by relentless thrombo-inflammation, enabling heightened propensity for I/RI events such as stroke [7,8].

As observed with mice stimulated with LPS, STM had comparable cerebral responses in both arterioles and venules when exposed to light/dye thrombosis, supporting findings demonstrating that a pro-inflammatory phenotype exists within their cerebral microvasculature (as quantified by enhanced leukocyte–endothelial cell adhesion and increased ROS production [32,57]). AnxA1_Ac2-26_ treatment reduced cerebral thrombosis and the use of Fpr antagonists Boc2 and WRW4 confirmed that these protective effects were caused by receptor engagement of peptide AnxA1_Ac2-26_ to Fpr2/ALX. Furthermore, research from our laboratory (Ansari et al., 2020, under review) has also shown AnxA1_Ac2-26_ to reduce citrullinated histone-3 (H3Cit+)-rich neutrophil extracellular trap (NET) production in SCD. These findings (along with those from other groups [58,59]) are of clinical significance as NETs have been recognized as critical components for venous and arterial thrombosis and inhibition of pathological NET formation may be beneficial for thrombo-inflammatory events and disorders such as SCD [59]. Taken together, these results demonstrate the versatility and innovative approach of AnxA1_Ac2-26_ as a therapeutic compound for the resolution of thrombo-inflammation.

We uncovered that although the Fpr-pan antagonist Boc2 abrogated the effects of the peptide in arterioles, no statistically significant effect was observed in venules irrespective of the thrombo-inflammatory environment, although there was a trend towards abrogation. The same could not be said for the Fpr2/ALX-specific antagonist WRW4, which was effective in annulling the peptide’s responses irrespective of vessel type or thrombo-inflammatory model. This dichotomy of behaviour of the peptide is less likely to be due to antagonist doses (as these were chosen based on dose–response curves) but could be due the fact that FPRs have a large number of diverse unrelated ligands that are able to bind to the receptor family to elicit pro- and anti-inflammatory effects that are specific to ligand and cell type. More recently, the concept of biased agonism has been coined to describe “the ability of a ligand to selectively activate subsets of downstream signalling pathways coupled to a receptor while inhibiting others” [60], which could help to explain differences observed between AnxA1_Ac2-26_ and co-administration with either the pan-antagonist Boc2 or the Fpr2-specific antagonist WRW4. Other factors that could be accountable for the variations in peptide and antagonist responses could be the known physiological differences between arterioles and venules e.g., shear rates (being predominantly higher in arterioles vs. venules) and differences in the thrombi formed in these respective vessels e.g., arteriolar thrombi being platelet-rich, but venular thrombi also containing leukocytes (typically near the surface of the microvascular thrombi) [61]. Despite these disparities, our data demonstrate the potent activity and versatility of AnxA1_Ac2-26_ to mitigate both LPS- and SCD-associated cerebral thrombosis (in both arterioles and venules) in an Fpr2/ALX-dependent manner. Correspondingly, whole protein AnxA1 has also been shown to afford protection against subsequent thrombotic events post stroke, demonstrating the diversity of AnxA1 and its mimetic peptide [29].

Platelets play a pivotal role in normal haemostasis and thrombus formation, and more recently have been found to also play a role in maintaining barrier function [6]. Once activated, platelets undergo a shape change to enhance adhesiveness, a process mediated by exposed glycoprotein receptors on the platelet surface [6]. GPVI is a unique platelet membrane glycoprotein whose binding with collagen (which is exposed on the extracellular matrix following stroke [6]) results in platelet activation and adhesion, and ultimately, thrombus formation. Platelets primarily rely on signalling through GPVI and C-type lectin-like type II transmembrane receptor (CLEC-2) to prevent bleeding [62]. GPVI, via inside-out signalling, enhances the affinity of integrins such as αIIbβ3 (the most abundant platelet receptor (80,000–100,000 copies per platelet) and necessary for aggregation), leading to platelet adhesion [53]. As such, given the important role that GPVI plays in thrombosis, we used flow cytometry to investigate whether the anti-thrombotic effects of AnxA1_Ac2-26_ involved GPVI signalling. By directly activating the GPVI pathway using CVX, we were able to discern that the AnxA1 peptide was able to decrease α_IIb_β_3_ activation and the surface expression of P-selectin, which in turn inhibits platelet activation. Interestingly, we have previously found that the parent protein does not reduce P-selectin expression in thrombin-stimulated platelets [29]. One might speculate that these discrepancies may lie in the different platelet stimuli and/or the downstream responses elicited by the binding of either the whole protein or the peptide to Fpr2/ALX and subsequent conformational changes of the receptor [26]. Equally, these effects may simply relate to the varied regulatory functions afforded by the peptide during the host defence response. Further experiments will help to tease out these mechanisms.

Other regulatory mechanisms (such as those involving cyclooxygenase 2 (‘COX2’)/hydroxyeicosatetraenoic acid-5 (HETE-5)/Lipooxygenase) may also be involved in the role that the AnxA1/Fpr2/ALX pathway plays in thrombo-inflammation. AnxA1 (and its mimetic peptide AnxA1_Ac2-26_), Lipoxin A_4_, and ATL (produced after aspirin acetylation of inducible COX-2) all exert their protective effects through FPR2/ALX at different phases of the inflammatory response. Phospholipase (e.g., calcium-dependent cytosolic phospholipase A_2_ (“cPLA_2_”)) activity releases arachidonic acid (“AA”) from phospholipids in the outer nuclear membrane. Once released, the free fatty acid can be metabolized *via* enzymatic pathways including the (COX) and lipoxygenase (LOX) pathways, generating 2-series prostaglandins (PGs) and thromboxanes (Txs) (COX pathway) or 4-series leukotrienes (LTs) and hydroxyeicosatetraenoic acids (HETEs) (LOX pathway) [63]. Transcellular production of lipoxins and leukotrienes ensues when contact is made between e.g., neutrophils and platelets. Previous research from our laboratory has shown that when aspirin is administered to mice with experimental stroke, they can produce ATL, which triggers pro-resolving responses through the engagement of the AnxA1/Fpr2/ALX pathway [17]. Furthermore, ASA administered to healthy volunteers is also capable of producing bioactive levels of ATL [64]. More recently, Sanches et al. showed that macrophages lacking AnxA1 have increased AA metabolism and eicosanoid production. This lack of AnxA1 favours LPS “over-priming” and exacerbated NLR Family Pyrin Domain Containing 3 (“NLRP3”) activation, demonstrating an important role for AnxA1 in inflammasome activation [63]. Other studies have also shown AnxA1 to exert its effects via the FPR2/ALX/p38 mitogen-activated protein kinase (“MAPK”)/COX-2 pathway in an experimental model of intracerebral haemorrhage, although other MAPKs may also be involved [65].

Patients with thrombo-inflammation (including stroke, sepsis, and SCD) all present with abnormal circulating platelet–leukocyte aggregates (with platelets binding to neutrophils in a P-selectin-dependent mechanism, an interaction that primes the leukocyte and promotes integrin activation), increasing their propensity to develop disseminated intravascular coagulation [49]. Studies have indicated that ischaemic stroke is not simply mediated by platelet aggregation, but also by other intravascular cells including neutrophils, although it remains unclear whether the direct interaction between platelets and neutrophils is critical for the pathogenesis of ischaemic stroke [59]. Von Brühl et al. demonstrated leukocytes (predominantly neutrophils) crawl and adhere to the endothelium, initiating and propagating venous thrombosis [66]. Additionally, neutrophils have now been shown to be involved in arterial thrombosis as well [67], although platelet activation and aggregation is the main driving factor. Furthermore, several distinct mechanisms have been postulated for neutrophil involvement in thrombosis including: the transfer of tissue factor from neutrophils to platelets, inducing thrombosis [68]; the release of specific mediators that affect thrombosis, including cathepsin G and neutrophil elastase (which inactivate anticoagulant systems such as tissue factor pathway inhibitor, thrombomodulin, and antithrombin), neutrophil oxidants (which e.g., inactivate thrombomodulin and ADAMTS13), and micro-RNAs [59]; and the generation of NETs which can trap and activate platelets via histone production [69]. Of interest, more recently, COVID-19 patients have been shown to have increased serum markers of NETs including myeloperoxidase-DNA (MPO-DNA) and H3Cit. Given our recent findings (Ansari et al., 2020, under review) that AnxA1_Ac2-26_ is able to reduce H3Cit^+^-rich NET production, transforming neutrophil phenotype from pro-NETotic to pro-apoptotic (thereby driving thrombo-inflammation resolution in SCD), we are currently investigating whether these findings can be exploited to provide therapeutic value for COVID-19 patients [70].

## 5. Conclusions

In conclusion, we provide strong supporting evidence that AnxA1 mimetic peptide AnxA1_Ac2-26_ possesses an arsenal of immune responses extending beyond that of an anti-inflammatory (e.g., attenuation of leukocyte-platelet responses post stroke, reduction of lipopolysaccharide-induced leukocyte adhesion and migration) and pro-resolution mediator to also include a role as an anti-coagulant and anti-thrombotic agent. These combined effects make AnxA1_Ac2-26_ a promising therapeutic candidate for promoting resolution in the context of thrombo-inflammatory diseases/conditions.

## Supplementary Materials

The following are available online at https://www.mdpi.com/2073-4409/9/11/2473/s1, Figure S1: Effect of FPR antagonists. Mice (C57BL/6) were subjected to vehicle LPS (10 µg/mouse) for 2 h and treated with vehicle (saline), pan FPR antagonist Boc2 (10 µg/mouse) or FPR2/ALX antagonist WRW4 (55 µg/mouse) 20 min prior to light/dye-induced thrombus formation, with time of onset and blood flow cessation times recorded for cerebral (A) arterioles and (B) venules. Data are means ± SEM of 5–6 mice/group. ^$$$$^ *p* < 0.0001 vs. same group for onset time, Figure S2: Onset and blood flow cessation times in mice with/without endotoxaemia. C57BL/6 mice or sickle cell transgenic mice (STM) were subjected to vehicle (saline) or LPS (0.4 mg/kg) for 2 h. A cranial window was performed, and FITC-dextran injected (10 mg/kg of 5%).Mice were then subjected to intravital microscopy and light/dye-induced thrombus formation, with time of onset and blood flow cessation times recorded for cerebral (A) arterioles and (B) venules. Data are means ± SEM of 5–6 mice/group. * *p* < 0.05, ** *p* < 0.01 vs. C57BL/6 saline group. ^##^ *p* < 0.01 vs. STM saline group. ^$^ *p* < 0.05, ^$$^ *p* < 0.0001 vs. same group for onset time, Figure S3: AnxA1_Ac2-26_ did not affect neutrophil counts in LPS-treated mice. Peripheral blood neutrophil counts were assessed following saline (vehicle) or AnxA1_Ac2-26_ (4 mg/kg) administration for 20 min following 2 h saline (vehicle) or LPS (0.4 mg/kg) administration. Data are means ± SEM of 5–6 mice/group. ** *p* < 0.01, *** *p* < 0.001 vs. LPS vehicle (saline), Figure S4: Effect of FPR antagonists in sickle cell transgenic mice (STM). STM were subjected to vehicle LPS (10 µg/mouse) for 2 h and treated with vehicle (saline), pan FPR antagonist Boc2 (10 µg/mouse) or FPR2/ALX antagonist WRW4 (55 µg/mouse) 20 min prior to light/dye-induced thrombus formation, with time of onset and blood flow cessation times recorded for cerebral (A) arterioles and (B) venules. Data are means ± SEM of 5–6 mice/group. ^$$$$^ *p* < 0.0001 vs. same group for onset time.
