# Supplementary material for: Targeting AnxA1/Formyl Peptide Receptor 2 Pathway Affords Protection against Pathological Thrombo-Inflammation
Source: Cells. 2020 Nov 13;9(11):2473. doi: 10.3390/cells9112473 (PMC7697101; doi:10.3390/cells9112473)
Supplement: Supplementary file 1 [file cells-09-02473-s001.pdf]

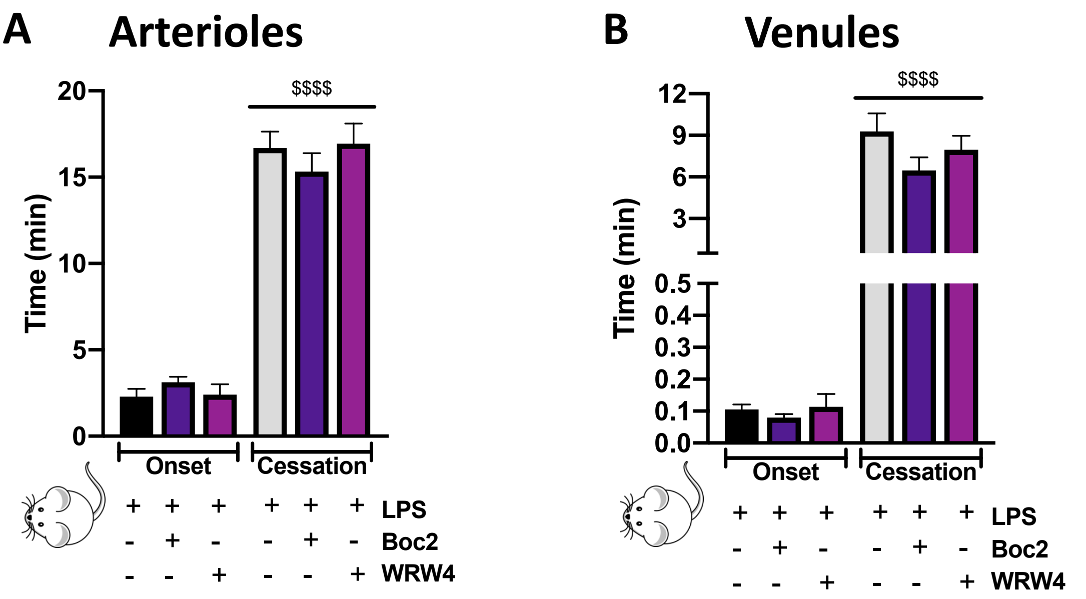

Figure S1. Effect of FPR antagonists. Mice (C57BL/6) were subjected to vehicle LPS (10 µg/mouse) for 2h and treated with vehicle (saline), pan FPR antagonist Boc2 (10 µg/mouse) or FPR2/ALX antagonist WRW4 (55 µg/mouse) 20 minutes prior to light/dye-induced thrombus formation, with time of onset and blood flow cessation times recorded for cerebral A) arterioles and B) venules. Data are means ± SEM of 5-6 mice/group. $^{\$\$\$\$}p < 0.0001$ vs. same group for onset time.

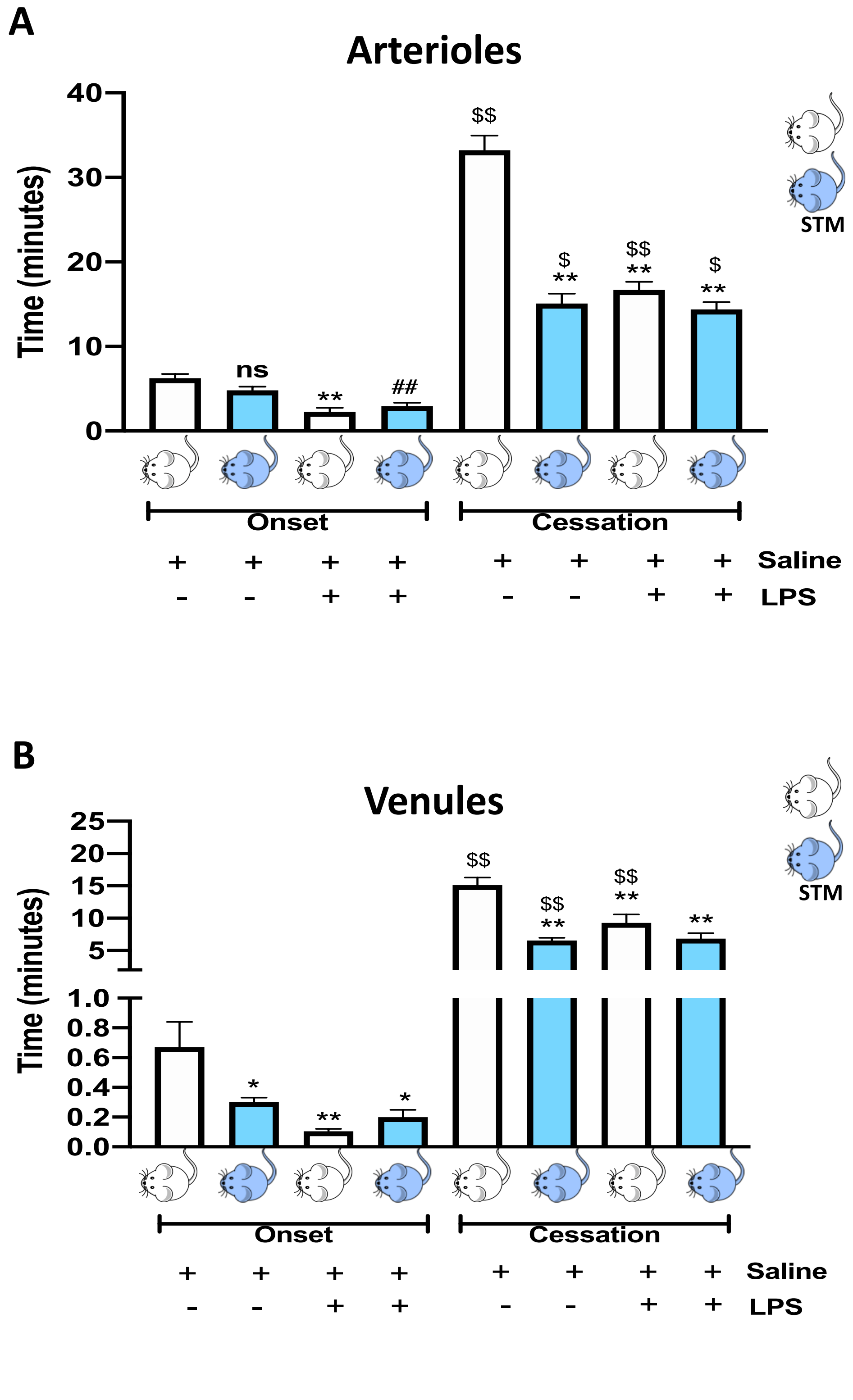

Figure S2. Onset and blood flow cessation times in mice with/without endotoxaemia. C57BL/6 mice or sickle cell transgenic mice (STM) were subjected to vehicle (saline) or LPS (0.4 mg/kg) for 2h. A cranial window was performed, and FITC-dextran injected (10 mg/kg of 5%). Mice were then subjected to intravital microscopy and light/dye-induced thrombus formation, with time of onset and blood flow cessation times recorded for cerebral (**A**) arterioles and (**B**) venules. Data are means ± SEM of 5-6 mice/group. $^{*}p < 0.05$, $^{**}p < 0.01$vs. C57BL/6 saline group. $^{\#\#}p < 0.01$ vs. STM saline group. $^{\$}p < 0.05$, $^{\$\$}p < 0.0001$ vs. same group for onset time.

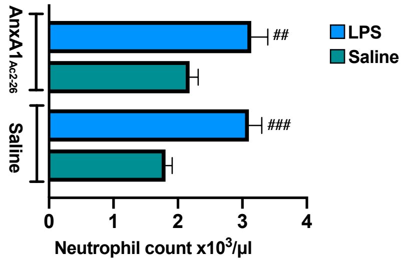

Figure S3. AnxA1$_{Ac2-26}$ did not affect neutrophil counts in LPS-treated mice. Peripheral blood neutrophil counts were assessed following saline (vehicle) or AnxA1$_{Ac2-26}$ (4 mg/kg) administration for 20 minutes following 2h saline (vehicle) or LPS (0.4 mg/kg) administration. Data are means ± SEM of 5-6 mice/group. ** $p < 0.01$, *** $p < 0.001$ vs. LPS vehicle (saline).

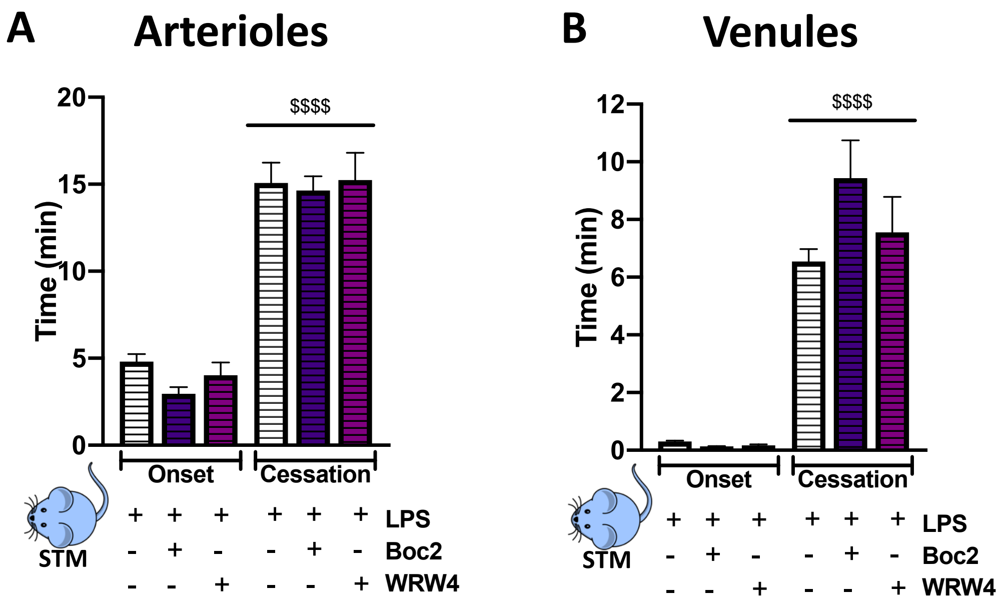

Figure S4. Effect of FPR antagonists in sickle cell transgenic mice (STM). STM were subjected to vehicle LPS (10 μg/mouse) for 2h and treated with vehicle (saline), pan FPR antagonist Boc2 (10 μg/mouse) or FPR2/ALX antagonist WRW4 (55 μg/mouse) 20 min prior to light/dye-induced thrombus formation, with time of onset and blood flow cessation times recorded for cerebral A) arterioles and B) venules. Data are means ± SEM of 5-6 mice/group. $^{\$\$\$\$}p < 0.0001$ vs. same group for onset time.
